# Assessment of COVID-19 risk factors of early and long-term mortality with prediction models of clinical and laboratory variables

**DOI:** 10.1186/s12879-024-09592-7

**Published:** 2024-07-09

**Authors:** Dawid Lipski, Artur Radziemski, Stanisław Wasiliew, Michał Wyrwa, Ludwina Szczepaniak-Chicheł, Łukasz Stryczyński, Anna Olasińska-Wiśniewska, Tomasz Urbanowicz, Bartłomiej Perek, Andrzej Tykarski, Anna Komosa

**Affiliations:** 1 Department of Hypertensiology, Angiology and Internal Medicine, Poznan University of Medical Sciences, Poznan, Poland; 2 Department of Cardiac Surgery and Transplantology, Chair of Cardio-Thoracic Surgery, Poznan University of Medical Sciences, ul. Długa 1/2, Poznan, 61-848 Poland

**Keywords:** Inflammation, LDH, COVID-19, Pneumonia

## Abstract

**Background:**

Coronavirus disease (COVID-19) may lead to serious complications and increased mortality. The outcomes of patients who survive the early disease period are burdened with persistent long-term symptoms and increased long-term morbidity and mortality. The aim of our study was to determine which baseline parameters may provide the best prediction of early and long-term outcomes.

**Methods:**

The study group comprised 141 patients hospitalized for COVID-19. Demographic data, clinical data and laboratory parameters were collected. The main study endpoints were defined as in-hospital mortality and 1-year mortality. The associations between the baseline data and the study endpoints were evaluated. Prediction models were created.

**Results:**

The in-hospital mortality rate was 20.5% (*n* = 29). Compared with survivors, nonsurvivors were significantly older (*p* = 0.001) and presented comorbidities, including diabetes (0.027) and atrial fibrillation (*p* = 0.006). Assessment of baseline laboratory markers and time to early death revealed negative correlations between time to early death and higher IL-6 levels (*p* = 0.032; Spearman rho − 0.398) and lower lymphocyte counts (*p* = 0.018; Pearson r -0.438). The one-year mortality rate was 35.5% (*n* = 50). The 1-year nonsurvivor subgroup was older (*p* < 0.001) and had more patients with arterial hypertension (*p* = 0.009), diabetes (*p* = 0.023), atrial fibrillation (*p* = 0.046) and active malignancy (*p* = 0.024) than did the survivor subgroup. The model composed of diabetes and atrial fibrillation and IL-6 with lymphocyte count revealed the highest value for 1-year mortality risk prediction.

**Conclusions:**

Diabetes and atrial fibrillation, as clinical factors, and LDH, IL-6 and lymphocyte count, as laboratory determinants, are the best predictors of COVID-19 mortality risk.

## Background

The coronavirus disease (COVID-19) pandemic, caused by severe acute respiratory syndrome coronavirus-2 (SARS-CoV-2), is the most unpredictable and overwhelming experience of the 21st century for health-care systems worldwide.

The clinical manifestations of COVID-19 infection vary from asymptomatic to pneumonia, which may lead to acute respiratory distress syndrome (ARDS), multiorgan failure and death [1]. Algorithms for the management of patients with COVID-19 and various therapeutic treatment regimens have been developed [2, 3]. Moreover, predictors of short- and long-term outcomes have been meticulously investigated to establish clinical factors and laboratory parameters that can differentiate patients with worse survival. Older age, diabetes, hypertension, and chronic kidney disease [4–7] have been reported to be strong predictors of mortality and morbidity due to respiratory deterioration. The role of laboratory parameters in predicting the severity of COVID-19 infection and its outcome is still a subject of discussion and remains unclear [8–13]. The available scientific data and our experience suggest that the assessment of baseline levels of laboratory markers together with further monitoring of their changes might be of great assistance for clinicians in terms of predicting disease severity, disease evolution and patient prognosis [14–18]. Not uncommonly, COVID-19 is characterized by acute and unpredictable deterioration and high mortality in certain groups of patients [19], particularly depending not only on the virus type. The course of the disease may be rapidly changing, which can often overload the medical system. Therefore, the identification of early predictors of deterioration and mortality is beneficial [20]. The rapid and enormous extent of the pandemic has forced the engagement of large amounts of medical resources; thus, knowledge of the disease and experience in its management has continuously increased with the implementation of sufficient diagnostics and therapies. However, even greater awareness of the outcomes of patients who survive the early disease period and are discharged from intensive care units is currently needed since the burden of persistent post-COVID-19 symptoms (long COVID-19 syndrome) and increased long-term morbidity and mortality have been widely highlighted [21–24]. The investigation of predictors of disease progression is of utmost importance.

The aim of our study was to determine which baseline parameters may provide the best prediction of early and long-term outcomes.

## Methods

### Study population

The study population included 141 COVID-19 patients (median (Q1; Q3) age 66 (53; 76) years, 72 males (51%) who were hospitalized at Poznan Temporary Hospital from the 5th of November to the 31st of December 2021).

The criteria for hospitalization included a positive COVID-19 Ag Rapid Test– Abbott Panbio result accompanied by symptoms of respiratory tract infection with at least one episode of desaturation (defined as saturation lower than 94%) or dyspnea on admission. Demographic and clinical data were collected. The baseline characteristics of the patients are shown in Table 1.

Exclusion criteria included rapid deterioration at admission leading to early death without collection of blood samples.

### Laboratory parameters

All analysed blood samples were collected from each patient at baseline (i.e. within the first 3 h from admission) at the same study point to assess simple blood morphology, biochemical parameters (including creatinine, urea, lactate dehydrogenase (LDH), aspartate aminotransferase (AST), alanine aminotransferase (ALT), C-reactive protein (CRP), procalcitonin, troponin, glucose, and electrolytes), lipid profiles, and coagulation parameters (including D-dimer (DD) and prothrombin time). Interleukin-6 (IL-6) levels were measured with the use of an enzyme-linked immunosorbent assay. Moreover, blood sampling was systematically repeated to monitor the disease course in a planned manner or additionally if deterioration occurred.

Chest computed tomography (CT) covering the apex to the lung base was performed for each patient to assess the presence and distribution of parenchymal lung tissue abnormalities. Electrocardiograms to assess heart rhythm and echocardiography in suspicion of heart failure or acute coronary syndrome were performed.

### Study endpoints

The main study outcomes were in-hospital mortality, death in in-hospital survivors at the 1-year follow-up and 1-year mortality. Additional study endpoints included inflammatory involvement of at least 50% of the lung parenchyma estimated on chest CT and significant clinical deterioration (in-hospital need for noninvasive or invasive ventilation or in-hospital death). We conducted a 12-month follow-up analysis following the discharge of each patient from the hospital. Telephone calls were made (3 attempts to access each phone number provided by the patient) to gather information regarding their current health status. Data concerning mortality were collected from the national database.

### Statistical analysis

Continuous variables were tested for a normal distribution using the Shapiro‒Wilk test and are reported as medians and interquartile ranges (Q1; Q3) since the data did not follow a normal distribution. Comparisons were performed by means of the Mann‒Whitney test. For the prediction analyses, the laboratory findings were divided by quartiles. For the parts of the study population with values of each aforementioned laboratory parameter below the 1st (low) and above the 3rd (high) quartiles, the predictors of each outcome were calculated. The chi-squared test was used for categorical data. The Spearman or Pearson (where relevant for nonparametric and parametric data) correlation coefficient was used for assessment of associations between variables. Receiver operating characteristic (ROC) analysis was carried out to determine the best model for mortality prediction. Analysis was performed with Statistica, Tibco and JASP statistical software (JASP Team; 2023. Version 0.18.1). A P value lower than 0.05 was considered to indicate statistical significance.

## Results

### In-hospital mortality

The in-hospital mortality rate was 20.5% (*n* = 29). The median (Q1; Q3) time from admission to early death was 15 (8; 19) days. Compared with survivors, nonsurvivors were significantly older (*p* = 0.001), were burdened with comorbidities, including diabetes (0.027) and atrial fibrillation (*p* = 0.006), and presented lower saturation at admission (*p* = 0.015) (Table 1).

**Table 1:** Demographic and clinical data in the whole study group and in subgroups divided based on early and long-term survival

Parameter	All patients (*n* = 141)	In-hospital survivors (*n* = 112)	In-hospital death (*n* = 29)	*p* value	Overall 1-year survivors (*n* = 91)	Overall 1-year nonsurvivors (*n* = 50)	*p* value
Age [years] Median (Q1; Q3)	66 (53;76)	64 (48.8; 74)	75 (63;85)	0.001	63 (47.5; 74)	73 (59.25; 81.75)	< 0.001
Sex Male/female (n,%)	72 (51%)/69 (49%)	50 (52.7%)/53 (47.3%)	13 (44.8%)/16 (55.2%)	0.451	49 (53.9%)/42 (46.2%)	23 (46%)/27 (54%)	0.373
Arterial hypertension (n,%)	78 (55.3%)	59 (52.7%)	19 (65.5%)	0.215	43 (47.3%)	35 (70%)	0.009
Diabetes (n,%)	40 (28.4%)	27 (24.1%)	13 (44.8%)	0.027	20 (22%)	20 (40%)	0.023
Ischaemic heart disease (n,%)	22 (15.6%)	15 (13.4%)	4 (13.8%)	0.955	13 (14.3%)	9 (18%)	0.630
History of myocardial infarction (n,%)	17 (12.1%)	12 (10.7%)	5 (17.2%)	0.336	10 (11%)	7 (14%)	0.599
History of stroke or TIA (n,%)	17 (12.1%)	11 (9.8%)	6 (20.7%)	0.119	9 (9.9%)	8 (16%)	0.293
Atrial fibrillation (n,%)	21 (15%)	12 (10.7%)	9 (31%)	0.006	9 (9.9%)	12 (24%)	0.046
Active malignancy (n,%)	8 (5.7%)	6 (5.4%)	2 (6.9%)	0.668	2 (2.2%)	6 (12%)	0.024
COPD (n,%)	8 (5.7%)	6 (5.4%)	2 (6.9%)	0.668	5 (5.5%)	3 (6%)	1.000
Asthma (n,%)	8 (5.7%)	6 (5.4%)	2 (6.9%)	0.668	6 (6.6%)	2 (4%)	0.712
Chronic kidney disease (n,%)	8 (5.7%)	4 (3.6%)	4 (13.8%)	0.056	3 (3.3%)	5 (10%)	0.132
Peripheral artery disease (n,%)	4 (2.8%)	3 (2.7%)	1 (3.5%)	1.000	1 (1.1%)	3 (6%)	0.128
Vaccinated against COVID-19 (n,%)	37 (26%)	30 (27.5%)	7 (24.1%)	0.816	19 (21.4%)	18 (36.7%)	0.051
SBP at admission (mmHg) median (Q1; Q3)	133 (120, 145)	135 (122;145.25)	125 (107; 141)	0.059	135 (122; 146)	129.5 (112; 143)	0.074
DBP at admission (mmHg) median (Q1; Q3)	72 (65, 80)	72 (66; 82.25)	73 (65; 80)	0.338	74 (66.5; 84)	71 (64.25; 78)	0.058
Saturation at admission (%) median (Q1; Q3)	95 (92.2, 96)	95 (93 ; 96)	94 (90; 95)	0.015	95 (93; 97)	94 (92; 95)	0.039
HR at admission (beats/min) median (Q1; Q3)	87 (76; 100)	87.5 (77; 100)	86 (75; 95)	0.384	90 (76.5; 101)	85.5 (76.25; 95.75)	0.376
IMPROVE-VTE risk score median (Q1; Q3)	2 (1; 2)	1 (1; 2)	2 (1; 2)	0.026	1 (1; 2)	2 (1; 2)	0.013
Daily dose of LMWH median (Q1; Q3)	80 (40–160)	40 (40; 160)	120 (70; 160)	0.004	NA	NA	NA
DOAC (n,%)	10 (7.1%)	7 (6.3%)	3 (10.3%)	0.429	NA	NA	NA

Abbreviations: COPD – chronic obstructive pulmonary disease, CRP – C-reactive protein, DOAC – direct acting oral anticoagulants, DD – D-dimer, DBP – diastolic blood pressure, IL-6 – interleukin – 6, LDH – lactate dehydrogenase, LMWH – low-molecular-weight heparin, Q1 – first quartile, Q3 – third quartile, SBP – systolic blood pressure, TIA – transient ischaemic attack, WBC – white blood cell count

Assessment of baseline laboratory markers and time to early death revealed negative correlations between time to early death and higher IL-6 levels (*p* = 0.032; Spearman rho − 0.398) and lower lymphocyte counts (*p* = 0.018; Pearson r -0.438). There were no similar correlations for CRP, LDH or PCT.

### Additional in-hospital study outcomes

In-hospital significant clinical deterioration was noted in 29.8% of patients (*n* = 42). Twelve patients required intubation and mechanical ventilation, 7 who died during hospitalisation and 5 who survived. Eight patients were transferred to the intensive care unit after deterioration, and seven were transferred to cardiac or pulmonary departments for further treatment.

Involvement of at least 50% of the lung parenchyma in the baseline chest CT scan was observed in 29.8% of patients (*n* = 42), 45.2% (*n* = 19) of whom experienced deterioration, 41.4% (*n* = 12) of whom died during hospitalization and 33.3% (*n* = 7) who died after discharge during the 1-year follow-up.

Patients’ treatment was based on the most current recommendations and anticoagulation was adjusted to patients’ weight and thrombosis risk (Table 1). Data were presented with the use of IMPROVE-VTE risk score. All patients were recommended to use low molecular weight heparin (LMWH) at treatment doses adjusted to the individual weight up to 10 to 20 days after the hospitalization (depending on the assessed severity of disease and risk related to co-morbidities).

### Overall 1-year mortality and mortality in survivors at discharge

Twenty-one patients died after discharge from the hospital during the follow-up period. Overall, the 1-year mortality rate was 35.5% (*n* = 50). The 1-year nonsurvivor subgroup was older (*p* < 0.001) and had more patients with arterial hypertension (*p* = 0.009), diabetes (*p* = 0.023), atrial fibrillation (*p* = 0.046) and active malignancy (*p* = 0.024) than did the survivor subgroup.

#### Comparison between 1-year survivors and nonsurvivors

Baseline laboratory findings differed significantly between patients with and without any study endpoints and between survivors and nonsurvivors in terms of early and long-term follow-up. (Table 2).

**Table 2:** Laboratory data were collected for patients without any study endpoint, with any study endpoint, in-hospital survivors, in-hospital nonsurvivors, nonsurvivors after discharge, overall 1-year survivors and nonsurvivors

Parameter	All patients (*n* = 141)	without study endpoints (*n* = 65)	any endpoint (*n* = 76)	*p* value	In-hospital survivors (*n* = 112)	In-hospital death (*n* = 29)	*p* value	Nonsurvivors after discharge (*n* = 21)	Overall 1-year survivors (*n* = 91)	Overall 1-year nonsurvivors (*n* = 50)	*p* value
WBC [x10e9/L] median (Q1; Q3)	6.37 (4.73; 8.28)	5.95 (4.39; 6.95)	6.98 (5.29; 8.95)	0.006	6.4 (4.95;7.9)	6.16 (4.23;8.62)	0.886	7.53 (5.31; 8.91)	6.35 (4.82; 7.58)	6.65 (4.77; 8.84)	0.41
Neu [x10e9/L] median (Q1; Q3)	4.83 (3.37; 6.65)	4.28 (3.06; 5.66)	5.45 (4.3; 7.47)	< 0.01	4.81 (3.45; 6.59)	5.14 (3.28; 7.92)	0.603	6.13 (4.21;7.29)	4.67 (3.24; 6.310	5.43 (3.40; 7.52)	0.097
Lymph [x10e9/L] median (Q1; Q3)	0.77 (0.56; 1.09)	0.82 (0.62; 1.39)	0.72 (0.53; 0.97)	0.025	0.84 (0.62;1.17)	0.59 (0.44;0.800	< 0.01	0.73 (0.53; 1.01)	0.85 (0.63; 1.26)	0.61 (0.49; 0.94)	0.001
PLT [x10e9/L] median (Q1; Q3)	194 (1160; 255)	187 (157; 236)	208 (162.5; 263.3)	0.215	199.5 (162.5; 2580	183 (144;223)	0.247	233 (174; 271)	199 (160.5; 248.5)	188.5 (159.5; 259.5)	0.936
MPV [fl] median (Q1; Q3)	9.5 (8.8; 10.2)	9.6 (8.9;10.4)	9.4 (8.7;10.2)	0.290	9.5 (8.7; 10.23)	9.5 (9; 10.20)	0.651	9.1 (8.7; 9.8)	9.6 (8.8; 10.4)	9.4 (8.8; 10.1)	0.444
AST [U/L] median (Q1; Q3)	48 (32.5; 69.5)	43 (30.8; 65.3)	52 (39; 70.5)	0.097	46 (31;69)	58 (42.5;80.75)	0.067	48 (25; 69)	45.5 (32.25; 64)	55 (35; 710	0.304
ALT [U/L] median (Q1; Q3)	33 (24; 52)	33 (25; 49)	33 (22;62)	0.739	33 (24;52.25)	33 (22;48)	0.939	31 (13;43)	33 (24.5; 53.5)	32.5 (18.25; 45.5)	0.168
Creatinine [umol/L] median (Q1; Q3)	83 (68.8; 117.5)	83 (70; 103)	83 (67.5; 121)	0.808	80 (66.75; 103)	98 (80.25; 133.75)	0.003	78 (63; 103)	82 (67; 101.5)	91 (70; 127)	0.087
LDH [U/L] median (Q1; Q3)	393 (276; 497)	326 (241; 443)	456 (329; 541)	< 0.001	387.5 (261.75; 483.5)	1254 (833; 2916)	0.023	482 (321; 530)	364.5 (254.75; 466.25)	483 (320; 551)	0.003
CRP [mg/L] median (Q1; Q3)	81 (44; 134)	56 (31; 100)	109.5 (54.5; 161.5)	< 0.001	85 (42.75;134.5)	76 (45;128)	0.83	130 (87; 192)	73 (37.5; 121)	100.5 (55.25; 148.75)	0.009
IL-6 [pg/mL] median (Q1; Q3)	30.4 (12.5; 66.2)	20.4 (8.2; 38.1)	54.7 (23.1; 111.2)	< 0.001	27.8 (10.3; 61.7)	47.3 (26.1; 126)	0.014	61.7 (36.8; 151)	23.8 (9.65; 56.4)	56.1 (27; 127.05)	< 0.001
Procalcitonin [ng/mL] median (Q1; Q3)	0.11 (0.07; 0.23)	0.09 (0.04;0.14)	0.16 (0.09;0.32)	< 0.001	0.09 (0.06; 0.18)	0.21 (0.15; 0.93)	< 0.001	0.18 (0.11; 0.40)	0.09 (0.05; 0.15)	0.19 (0.13; 0.61)	< 0.001
DD [ng/mL] median (Q1; Q3)	1000 (584; 1743)	820 (444; 1359)	1075.5 (660.8; 1893.5)	0.053	873.5 (525.25; 1458)	1254 (833; 2916)	0.01	1097 (711; 1734)	859 (501; 1333.5)	1196 (752; 2508.75)	0.004

Abbreviations: ALT – alanine aminotransferase, AST – aspartate aminotransferase, CRP – C-reactive protein, LDH – lactate dehydrogenase, DD – D-dimers, IL-6 – interleukin – 6, Lymph– lymphocyte count, MPV – mean platelet volume, Neu – neutrophil count, PLT – platelet count, Q1 – first quartile, Q3 – third quartile, WBC – white blood cell count

### Prediction of COVID-19-related mortality and morbidity

The baseline laboratory parameters differed significantly between the survivors and nonsurvivors at both the in-hospital and 1-year follow-ups (Table 2). Among all analysed markers, lymphocytes, Il-6 and LDH, procalcitonin and CRP were significantly different in both early and long-term observation. The details of the prediction analysis are outlined in Table 3.

**Table 3:** Prediction analyses of in-hospital and 1-year mortality according to clinical and laboratory factors

	In-hospital mortality Univariable analysis	*p*	1-year mortality Univariable analysis	*p*
Sex (female)	OR 1.370 95% CI 0.603–3.112	0.452	OR 1.370 95%CI 0.685–2.737	0.373
Age	OR 1.047 95%CI 1.016–1.078	0.003	OR 1.040 95%CI 1.016–1.065	0.001
Saturation at admission	OR 0.975 95%CI 0.953–0.997	0.027	OR 0.986 95%CI 0.965–1.007	0.179
Arterial hypertension	OR 1.707 95%CI 0.729–3.997	0.218	OR 2.605 95%CI 1.253–5.413	0.010
Diabetes	OR 2.558 95%CI1.093-5.986	0.030	OR 2.367 95%CI 1.115–5.023	0.025
COPD	OR 1.309 95%CI 0.250–6.849	0.750	OR 1.098 95%CI 0.251–4.798	0.901
Coronary artery disease	OR 1.565 95%CI 0.552–4.441	0.400	OR 1.317 95%CI 0.520–3.339	0.562
Hyperlipideamia	OR 0.995 95%CI 0.399–2.482	0.992	OR 1.391 95%CI 0.651–2.973	0.394
Peripheral artery disease	OR 1.298 95%CI 0.130-12.955	0.824	OR 5.745 95%CI 0.581–56.756	0.135
Chronic kidney disease	OR 4.320 95%CI 1.011–18.464	0.048	OR 3.259 95%CI 0.745–14.257	0.117
Atrial fibrillation	OR 3.750 95%CI 1.395–10.078	0.009	OR 2.877 95%CI 1.117–7.410	0.029
History of stroke or TIA	OR 2.395 95%CI 0.803–7.145	0.117	OR 1.735 95%CI 0.624–4.824	0.291
White blood cells	OR 1.050 95%CI 0.940–1.172	0.386	OR 1.078 95%CI 0.977–1.189	0.134
Neutrophils	OR 1.085 95%CI 0.970–1.214	0.152	OR 1.118 95%CI 1.006–1.243	0.038
Lymphocytes	OR 0.107 95%CI 0.026–0.447	0.002	OR 0.191 95%CI 0.068–0.541	0.002
Monocytes	OR 0.258 95%CI 0.032-2.100	0.205	OR 0.355 95%CI 0.069–1.832	0.216
Platelets	OR 0.997 95%CI 0.992–1.002	0.233	OR 0.999 95%CI 0.996–1.003	0.722
Red blood cells	OR 0.426 95%CI 0.199–0.910	0.028	OR 0.363 95%CI 0.179–0.736	0.005
Interleukin 6	OR 1.003 95%CI 0.999–1.006	0.125	OR 1.011 95%CI 1.004–1.018	0.001
CRP	OR 1.001 95%CI 0.996–1.007	0.638	OR 1.006 95%CI 1.001–1.011	0.011
Procalcitonin	OR 1.204 95%CI 0.997–1.453	0.053	OR 3.403 95%CI 1.218–9.506	0.019
Creatinine	OR 1.013 95%CI 1.004–1.021	0.004	OR 1.010 95%CI 1.002–1.018	0.018
LDH	OR 1.003 95%CI 1.001–1.005	0.013	OR 1.003 95%CI 1.001–1.005	0.003
D dimers	OR 1.000 95%CI 1.000–1.000	0.709	OR 1.000 95%CI 1.000–1.000	0.671

Abbreviations: COPD – chronic obstructive pulmonary disease, CRP – C-reactive protein, DD – D-dimer, DBP – diastolic blood pressure, IL-6 – interleukin – 6, LDH – lactate dehydrogenase, Q1 – first quartile, Q3 – third quartile, SBP – systolic blood pressure, TIA – transient ischaemic attack

Univariable and multivariable analyses were performed to reveal predictors of in-hospital and 1-year mortality.

Multivariable analysis for in-hospital mortality revealed significant differences in age (OR 1.075, 95% CI 1.010–1.143, *p* = 0.024), diabetes status (OR 18.359, 95% CI 2.772-121.604, *p* = 0.003), atrial fibrillation status (OR 7.382, 95% CI 1.157–47.097, *p* = 0.034), lymphocyte count (OR 0.003, 95% CI 0.000-0.067, *p* < 0.001), LDH levels (OR 1.012, 95% CI 1.005–1.018, *p* < 0.001), IL-6 levels (OR 1.010, 95% CI 1.001–1.019, *p* = 0.037), platelet count (OR 0.981, 95% CI 0.967–0.995, *p* = 0.0100), red blood cell count (OR 0.100, 95% CI 0.18–0.545, *p* = 0.008), CRP levels (OR 0.985, 95% CI 0.972–0.999, *p* = 0.030), and red blood cell count (OR 0.100, 95.

Multivariate analysis of 1-year mortality revealed that diabetes (OR 7.031, 95% CI 2.193–22.542, *p* = 0.001), atrial fibrillation (OR 8.217, 95% CI 1.932–34.943, *p* = 0.004), laboratory data lymphocyte count (OR 0.041, 95% CI 0.007–0.252, *p* < 0.001), red blood cell count (OR 0.167, 95% CI 0.056–0.492, *p* = 0.001), IL-6 level (OR 1.012, 95% CI 1.004–1.020, *p* = 0.003) and LDH level (OR 1.005, 95% CI 1.002–1.009, *p* < 0.001) were predictive of mortality.

### The receiver operator curve (ROC)

**Fig. 1:**
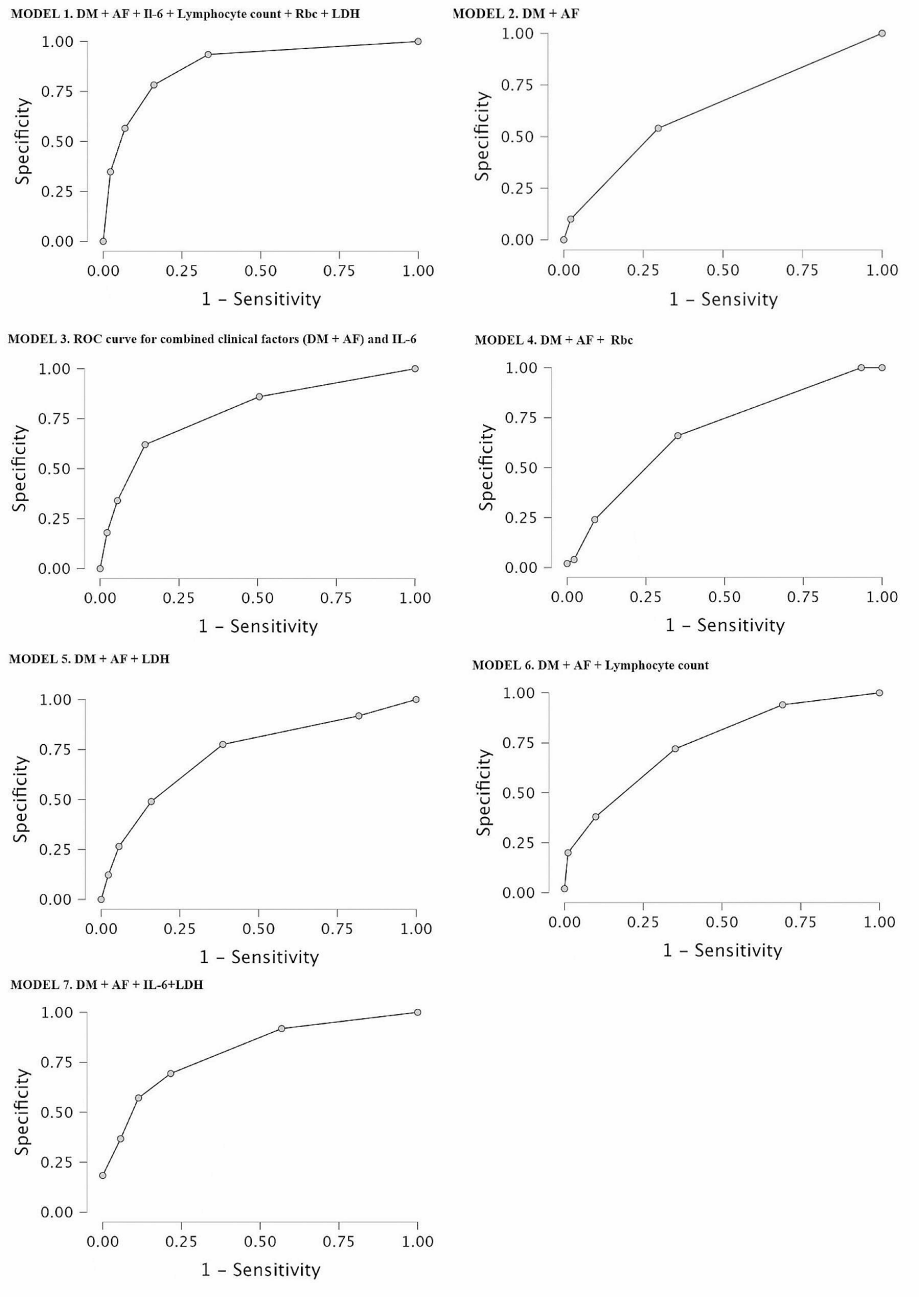
ROC analyses of the predictive value for 1-year all-cause mortality for different models composed of clinical and laboratory factors. Abbreviations: AF – atrial fibrillation, DM – diabetes mellitus, IL-6 – interleukin – 6, LDH – lactate dehydrogenase, Rbc – red blood cell count

Multivariate and ROC analyses revealed the predictive value for 1-year all-cause mortality as a multifactorial model including clinical factors (diabetes mellitus and atrial fibrillation) followed by laboratory parameters (lymphocyte count, Rbc, LDH and Il-6), yielding a sensitivity of 65.2% and specificity of 90.8% and an area under the curve (AUC) of 0.897, as shown in MODEL 1 in Fig. 1.

Due to the complexity of the presented model, we performed receiver operating characteristic (ROC) analysis for mortality prediction based on clinical factors (DM and AF), which revealed an area under the curve of 0.630, a sensitivity of 24.0% and a specificity of 90.1%, as shown in Fig. 1.

Due to the insufficient accuracy of ROC analysis based solely on clinical factors, the laboratory parameters were incorporated in a stepwise manner. ROC analysis revealed the highest accuracy when clinical factors were combined with the IL-6 concentration, yielding a sensitivity of 40.0%, a specificity of 90.1% and an area under the curve (AUC) of 0.800, as shown in MODEL 3 in Fig. 1. The combination of clinical factors and Rbc revealed an area under the curve of 0.679, yielding a sensitivity of 28.0% and a specificity of 87.9%, as shown in MODEL 4 in Fig. 1. The ROC curve results for mortality prediction combining clinical factors with separate LDH and lymphocyte concentrations revealed areas under the curve of 0.728 (yielding a sensitivity of 40.8% and specificity of 88.6%) and 0.760 (yielding a sensitivity of 38.0% and specificity of 87.9%), respectively, as presented in MODEL 5 and 6 in Fig. 1.

Finally, ROC analysis based on clinical factors and a combination of two laboratory parameters was performed, and a statistically significant model was reached when clinical factors (DM and AF) were combined with laboratory parameters such as lymphocyte count and IL-6, yielding a sensitivity of 64%, a specificity of 87.9% and an area under the curve (AUC) of 0.818, as shown in Model 7 in Fig. 1.

## Discussion

Our analysis showed that the combination of clinical and baseline laboratory data enables the most accurate prediction of mortality risk in patients with COVID-19 infection. The natural course of COVID-19 infection varies depending on the patient’s individual characteristics, such as age, comorbidities, and immune system status. Clinical variables, such as diabetes and hypertension, have been reported to be important factors influencing the course of COVID-19 [25]. In our analysis, we confirmed the significance of diabetes and atrial fibrillation on patient outcomes. Early on, diabetes was recognized as an important factor contributing to disease severity and mortality and a greater risk of respiratory complications. Furthermore, the newest studies [26] suggest a relationship between COVID-19 and new-onset diabetes. Chronic or de novo AF has been associated with a worse in-hospital prognosis, a greater complication rate and increased utilization of healthcare resources, both in COVID-19 and non-COVID-19 circumstances [27–30]. A severe course of COVID-19 observed in some patients may lead to death or long-lasting complications. The mortality rate due to COVID-19 infection therefore differs depending on several determinants [31].

Several studies have investigated the role of various biomarkers in the evaluation of mortality risk in terms of the escalation of respiratory support [32] and complication rate [33]. Careful evaluation of laboratory parameters may be helpful for estimating the severity of infection and patient prognosis [34, 35]. Here, we presented the distribution of laboratory markers of inflammation and analysed the relationships between blood sample results and short- and long-term outcomes. The most frequent laboratory deviations in patients with COVID-19 infection are lymphopenia, neutrophilia, thrombocytopenia, and elevated levels of serum C-reactive protein (CRP). Common abnormalities in hematological tests include increased ferritin levels, prolonged prothrombin times and elevated D-dimer levels [36]. Other inflammatory biomarkers, including CRP and IL-6, have been extensively investigated and used in daily in-hospital practice for patients with COVID-19 [31]. CRP is a widely used parameter for all types of infections and has high reliability. A high CRP level is associated with severe pneumonia in COVID-19 patients and is a predictor of deterioration to acute respiratory distress syndrome (ARDS) and death [37]. Our study demonstrated that simple whole-blood morphology analysis and LDH and IL-6 levels may also have additional predictive value for the determination of COVID-19 complications. Il-6 is a prototype cytokine and shows pleiotropic activity necessary for host defence [38]. It is rapidly and extensively produced in the course of tissue damage related to infection. It induces a large amount of inflammatory acute phase proteins and mediates a variety of signalling pathways, cell proliferation and apoptosis. However, abnormal Il-6 production may lead to deleterious effects. The level of IL-6 was found to be much greater in COVID-19 patients and correlated with disease severity [39].

LDH (lactate dehydrogenase) is an enzyme that plays an essential role in the process of intracellular energy production. It is most active in the liver, heart, kidneys, muscles, lungs, brain and red blood cells (erythrocytes); thus, it is not specific to any certain tissue. LDH elevation has been reported in cardiac ischemia, malignancies and other pathologies [40], as it is indicative of cellular damage and hypoxia. It is believed to be a convenient biomarker of the systemic state of hyperinflammation [41]. Fialek et al. [40] performed a meta-analysis of studies that showed the value of LDH as a biomarker for the determination of COVID-19 severity.

The D-dimer level is often significantly elevated in patients with COVID-19. However, in our group, we did not confirm that the D-dimer level was a predictor of study endpoints.

The clinical condition of COVID-19 patients often deteriorates rapidly as a consequence of hyperinflammation due to cytokine storms that can lead to multiorgan damage [42, 43]. It is critically important to identify factors that determine a worse prognosis and a greater mortality risk. Our analysis revealed that clinical determinants, including diabetes and atrial fibrillation, are crucial for mortality risk assessment. However, simple models based on clinical variables do not provide as much information as more extensive models composed of clinical and laboratory determinants. Therefore, we believe that examination of LDH, IL-6 and lymphocyte count is a valuable method and should be performed for each patient diagnosed with COVID-19 together with determination of the presence of comorbidities.

Study limitations: This study was performed on a population of patients during the COVID-19 pandemic. Currently, the World Health Organization (WHO) has announced that the COVID-19 epidemic has already ended. However, we observe that the disease is still present and may lead to an unpredictable course with various complications. Moreover, long-lasting complications are commonly reported, with higher mortality in patients who survived COVID-19 being the most serious complication. Therefore, we believe that the determination and use of predictive models are still crucial. The second limitation is that the number of analysed patients was relatively low, but all of them were treated at one centre by one team; therefore, any bias related to the different management and therapeutic methods was avoided. Due to dynamic changes in virus biology and the significant impact of vaccination on the disease course, no standard of care for COVID-19 patients has been established. Moreover, we are aware of the relatively low significance of laboratory data in terms of odds ratios; however, our extensive prediction models showed that adding laboratory parameters to clinical models provides a much more valuable prediction of 1-year mortality.

## Conclusions

Diabetes and atrial fibrillation are clinical factors, and IL-6 and lymphocyte count are laboratory determinants that provide the best predictive model for the assessment of COVID-19 mortality risk.

## Data Availability

The datasets used and analysed during the current study are available from the corresponding author on reasonable request.
